# Effects of Anatomical Variation on Ganglion Cell and Nerve Fibre Layer Evaluation by Optical Coherence Tomography

**DOI:** 10.3390/jcm13237193

**Published:** 2024-11-27

**Authors:** Sami Dabbah, Jakob Bjerager, Mohamed Belmouhand, Simon P. Rothenbuehler, Inger Christine Munch, Miriam Kolko, Michael Larsen

**Affiliations:** 1 Department of Ophthalmology, Rigshospitalet, 2100 Copenhagen, Denmark; jakob.kondras.bjerager.02@regionh.dk (J.B.); mohamed.belmouhand@regionh.dk (M.B.); lars.michael.larsen@regionh.dk (M.L.); 2 Faculty of Health and Medical Science, University of Copenhagen, 1172 Copenhagen, Denmark; 3 Department of Ophthalmology, Odense University Hospital, 5000 Odense, Denmark; 4 Department of Ophthalmology, University Hospital Basel, 4031 Basel, Switzerland; simon.rothenbuehler@usb.ch; 5 Centre for Clinical Research and Prevention, Bispebjerg and Frederiksberg Hospital, 2400 Frederiksberg, Denmark

**Keywords:** visual field prediction, retinal nerve fibre layer, retinal ganglion cell layer, retinal anatomy, optical coherence tomography, structural analysis, Hood Report, automated perimetry

## Abstract

**Background/Objectives**: The automated analyses of optical coherence tomography (OCT) scans of the retina occasionally suggest the presence of tissue deficits when no visual field defects can be detected. This study was made to find the sources of such alerts. **Methods:** Data from a population-based cohort of 360 participants aged 30–80 years was analysed for the anatomical sources of alerts after the extensive exclusion of participants where any suspicion of abnormality could be raised. An analysis was made of 12 × 9 mm volume scans centred between the disc and the fovea. The exclusions comprised 107 eyes with definite or borderline abnormal visual fields or other potentially confounding characteristics. A statistical analysis of the thickness patterns was made using the manufacturer’s proprietary algorithm. The analysis comprised alerts corresponding to local layer thickness values in the lower 5th percentile of an independent reference population. **Results:** Of the 613 eligible healthy eyes, thickness deficit alerts were seen in 391. They were related to the angle between the temporal nerve fibre ridges being wider, narrower, or rotated compared to the reference template in 174 eyes and to the variations in the size of the macula in 207 eyes. The source was unidentifiable in 28 eyes. The common sources were a thin papillomacular nerve fibre layer accompanied by arcuate nerve fibre ridges spaced far apart and a thinly, but wider than the normal macular ganglion cell layer. **Conclusions:** Anatomical variation in the retinal nerve fibre and ganglion cell layers was the source of more than 90% of the thickness deficit alerts in the eyes with normal visual fields.

## 1. Introduction

Glaucoma leads to the attenuation of the inner layers of the retina in concert with the disappearance of retinal ganglion cell bodies and their nerve fibres. This attenuation is challenging to evaluate using slit-lamp biomicroscopy; however, optical coherence tomography (OCT) offers potential advantages, particularly when reference scans from prior examinations are available for comparison and for identifying substance loss. In the absence of such reference scans, the assessment must rely on a template of a healthy retina for accurate evaluation. By subtracting the template from the sample, a pattern of structural deficits can be identified and subsequently transformed into the visual field domain. This process generates patterns of structural deviations that can alert clinicians to potential locations of visual field defects. In practice, the algorithms designed to translate retinal structural data into visual field alerts may occasionally indicate the presence of visual field defects that do not actually exist, resulting in what are referred to as false alerts. One potential solution to this issue is a didactic approach. It consists of underlining that the deviations between the template and sample are based on structure, not on function, and that their projection onto the visual field is made only for ease of interpretation. Another approach is to look for the anatomical explanation behind the differences between the template and the sample, something we have explored in this study.

## 2. Materials and Methods

### 2.1. Design

The study excluded all the cases with any suspicion of visual field defect, optic nerve damage, or glaucoma, meaning that all the structure-based alerts in the eyes eligible for analysis had no functional correlate and therefore were produced by the limitations of the method of imaging and analysis.

This was a cross-sectional observation study of 720 eyes in 360 participants of same-sex twins aged 18 years or older in self-reported good eye health. The participants were recruited from the Danish Twin Registry, a prospectively maintained database of all thet new-born twins in Denmark [1]. The study examinations were made between March 2019 and June 2020 at the Department of Ophthalmology of the Rigshospitalet in Copenhagen, Denmark. The raw data supporting the conclusions of this article will be made available by the authors on request.

The participants were interviewed about their medical history, family history, current use of medication and allergies. The ophthalmic examination included refractioning, an assessment of the best-corrected visual acuity (BCVA) using Early Treatment Diabetic Retinopathy Study (ETDRS) charts at 4 m, slit-lamp biomicroscopy, intraocular pressure (IOP) measured by rebound tonometry (iCare TAO1i, iCare USA, Raleigh, NC, USA), automated perimetry (Octopus 900, Dynamic 24-2 standard white/white/4000 visual field test, Haag Streit, Koeniz, Switzerland), fundus OCT (Triton SS-OCT, Topcon, Tokyo, Japan and Spectralis OCT 2, Heidelberg Engineering, Heidelberg, Germany), fundus autofluorescence photography (Spectralis OCT 2), axial length measurement (IOL Master 700, Carl Zeiss Meditech, Jena, Germany), and colour and red-free mydriatic fundus photography (TRC 50DX, Topcon, Tokyo, Japan).

Visual field examination to an eccentricity of 24 degrees was made before pupil dilation. Wide-field SS-OCT volume scans covered a fundus field of a 12 mm width and 9 mm height centred between the fovea and the optic disc and consisting of 256 parallel b-scans, each composed of 512 a-scans. Myopia mode was off. Five-field colour and red-free fundus photographs were centred on the fovea, the temporal macula, the optic disc, and the upper and lower vascular arcades.

**Figure 1:**
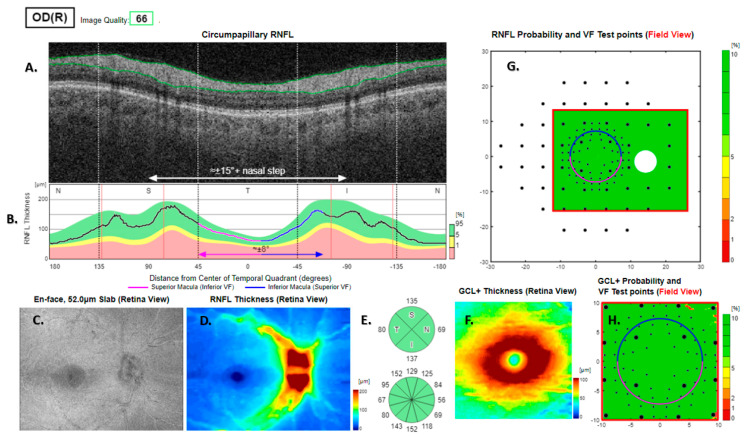
Volume scan 12 × 9 mm from a healthy right eye. The peripapillary retinal nerve fibre layer (RNFL (**A**)) and its thickness curve in ((**B**) black, blue and purple) falls entirely within the reference range of the (**B**) green area; N, nasal; S, superior; T, temporal; I, inferior quadrants; 0 degrees representing the temporal horizontal meridian. Red vertical lines (**B**) show the location of the superior and inferior arcuate trunk vessels. The peaks of the superior and inferior arcuate nerve fibre ridges or the template or reference curve are close to the innermost red lines. The nerve fibre ridges of the study eyes are close to those of the template. An en face slab of the inner retina shows an RNFL (**C**), a normal RNFL thickness map (**D**), and a normal RNFL sector analysis (**E**). The macular ganglion cell and inner plexiform layer (GC-IPL) thickness map is of normal thickness and shape (**F**). The RNFL- and GC-IPL thickness analyses do not suggest the presence of any statistically significant visual field (VF)-projected tissue defects ((**G**,**H**) green colour indicating that thickness is within the best 95% of healthy subjects at a given test location). GCL+ above = GC-IPL.

Optical coherence tomography scans were analysed using proprietary commercial automated segmentation and thickness pattern analysis software (Hood Report, IMAGEnet i-base version 3.23.0, Topcon, Tokyo, Japan). The output of the report included a display of the retinal nerve fibre layer (RNFL) thickness, a map of the combined retinal ganglion cell and inner plexiform layer (GC-IPL) thickness, and two accompanying visual field projections of the deviation from the normal thickness of the nerve fibre and ganglion cell layers (Figure 1). Thus, every location in the visual field was assigned a colour code value so that the maps displayed patterns of conformity and deviation from statistical normality. The colour code was optimised for the display local thickness in the lower 5% among healthy subjects. They should not be taken to represent the predicted visual field sensitivity [2].

Eyes were excluded if one of the following conditions were present: (1) a prior diagnosis of glaucoma made by an ophthalmologist, (2) structural fundus characteristics indicative of the potential presence of glaucoma made by slit-lamp biomicroscopy or any of the aforementioned imaging methods, (3) glaucoma suspecion based on visual field defects with corresponding abnormalities of the optic disc or the retinal nerve fibre layer, (4) an IOP higher than 25 mmHg, (5) insufficient OCT quality in the opinion of the reviewing physician, (6) static computerised perimetry (Octopus, Haag-Streit) with a mean defect (MD) > 6 dB, (7) false positive perimetry responses > 15%, (8) poor cooperation or examination fatigue noted by the examining investigator or reliability factor > 10%, (9) and any other eye disease or abnormality, including optic disc anomalies that could lead to incorrect retinal segmentation. The objective of the exclusion process was to restrict the analysis population to indisputably healthy eyes with normal vision.

The visual field-projected thickness deviation maps from Hood Report (Figure 1G,H) were examined by two investigators, Sami Dabbah (SD) and Michael Larsen (ML), and compared with the fundus anatomy patterns seen on OCT and fundus photography without reference to the corresponding perimetry data in the first round. The thickness deviation in the lower 5% of the reference population was considered a visual field-projected thickness deficit alert, seen as a colour change from green (no deviation from normal) to yellow, orange, or red (deviation). They were classified according to whether it was based on the analysis of the RNFL or the GC-IPL. A search for the anatomical patterns of variation that could hypothetically explain the deviation was inspired by Hong et al. [3], who described anatomical variations in the angle between the temporal nerve fibre ridges that accompany the upper and lower vascular arcades of the fundus. In the second round, tentative classification of the findings was drawn up after an initial round of inspection by SD and revised in collaboration with ML. Repeated deficit alerts in RNFL or GC-IPL with visually similar location, shape, and extent were set as a classification type, after which in a third round of all the classifications was made by SD. In the final round, all the types of RNFL and GC-IPL deficit alerts were compared to the visual field perimetry of the corresponding eye to exclude an absolute visual field defect.

The nerve fibre layer-derived maps and the ganglion cell-inner plexiform layer maps were considered independently. A comparison of the two modalities was made only after the completion of the separate analyses.

### 2.2. Statistical Analysis

Microsoft Excel 360 for Windows was used for demographic statistics, GraphPad Prism was used for the reproducibility of the measurements analyses, and R-Studio was used for all the other statistical analyses. The normality was tested by Shapiro–Wilk normality tests. The median and range was used to describe the study population characteristic. Parametric parameters were reported in the means and standard deviations (SDs) while non-parametric parameters were reported in the medians and inter-quantile ranges (IQRs).

The grade comparisons were evaluated using a conditional logistic regression model within a matched co-twin multivariable analysis framework. The model adjusted for the inclusion of either one or both twins in the analysis.

## 3. Results

Of the 720 eyes in the 360 twins, 613 eyes (85%) in 327 twins (91%) with a median age of 59 (range 30–80) years were eligible for analysis. The exclusions comprised 107 eyes (15%) in 74 participants (20%) with (moderate or advanced primary open-angle glaucoma (n = 6) glaucoma suspecion based on visual field defects with corresponding abnormalities of the optic disc or the retinal nerve fibre layer (n = 12), MD > 6 dB (n = 37) or false positive automated perimetry responses >15% (n = 13), IOP > 25 mmHg (n = 2), insufficient OCT quality (n = 18), poor general cooperation with a reliability factor >10 or examination fatigue (n = 9), and other ocular diseases (n = 10). In 41 twins (11%), only one eye was included. After exclusions, the median best-corrected visual acuity (BCVA) was 89 (range 70–100), and the Early Treatment Diabetic Retinopathy Study (ETRDS) letters and median value of the static computerised perimetry mean defect was 1.80 (range −1.50 to 5.90) dB (Table 1). All the eyes had BCVA 70 ETDRS letters (Snellen visual acuity 0.5) or better.

**Figure 2:**
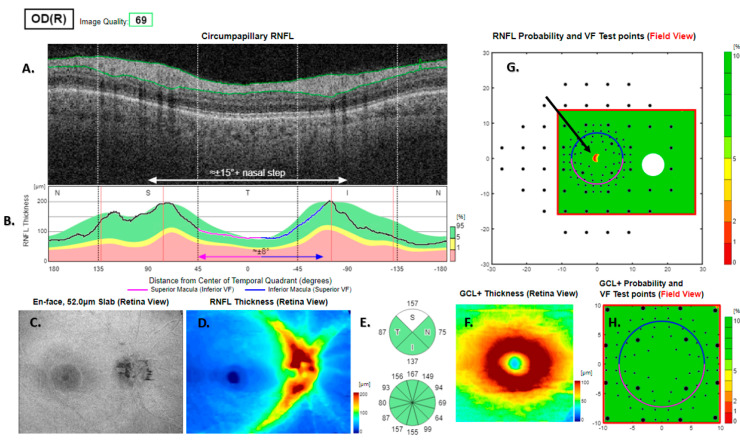
A red foveola RNFL-based deficit alert (black arrow in (**G**)) in a healthy eye. The peripapillary retinal nerve fibre layer (RNFL (**A**)) and its thickness curve in ((**B**) black, blue and purple); N, nasal; S, superior; T, temporal; I, inferior quadrants; 0 degrees representing the temporal horizontal meridian. Red vertical lines (**B**) show the location of the superior and inferior arcuate trunk vessels. The peaks of the superior and inferior arcuate nerve fibre ridges or the template or reference curve are close to the innermost red lines. An en face slab of the inner retina shows the RNFL analysis (**C**), RNFL thickness map (**D**), and RNFL sector analysis (**E**). The macular ganglion cell and inner plexiform layer (GC-IPL) thickness map is shown on (**F**). The RNFL probability map is shown on (**G**) and the GC-IPL probability map on (**H**). In (**G+H**) green colour indicates that thickness is within the best 95% of healthy subjects at a given test location. For other colours, see colour bar next to (**G+H**). VF, visual field. GCL+ above = GC-IPL.

In 447 (73%) of all the RNFL-based visual field projections, a small crescent or dot alert with a typical largest diameter of 1–3 degrees was shown at the centre of the visual field. This alert had no identifiable correlate in the peripapillary RNFL curves. It also, for methodological reasons, had no correlate in the perimetry data, because the centre location was used for the fixation target and therefore did not undergo examination. This alert was therefore omitted from further consideration and labelled the red foveola phenomenon (Figure 2).

The RNFL analysis was unremarkable in 289 (47%) of the eyes and the GC-IPL analysis was unremarkable in 368 (60%) of the eyes (Figure 1; Table 2). In 222 (36%) of the eyes, no RNFL- or GC-IPL-based alerts were found (Table 2). RNFL-based alerts comprised 5 archetypes defined by location, form, size, and number, occurring alone or in combination.

There was no limit to the number of alert-types that could be registered for a given eye.

**Figure 3:**
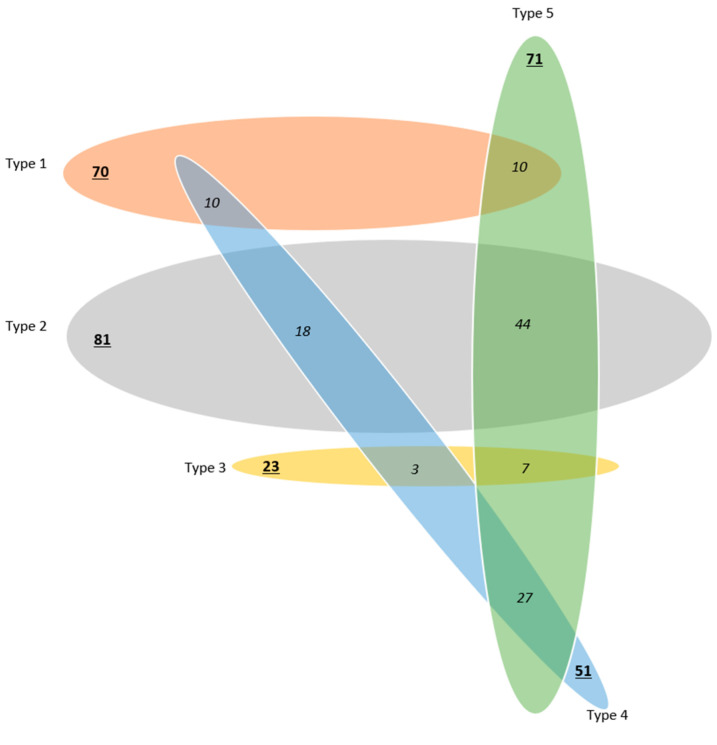
Combination diagram showing the degree of per-eye overlap between the RNFL-based visual field-projected tissue defect alerts. A total of 119 combinations were found. Types 1, 2 and 3 had 0 combinations. Types 4 and 5 had combinations with all the other groups. Type 5 and 2 were frequently combined, accounting for 60% of the type 5 combinations. The underlined numbers represent the total numbers of eyes per group.

In 324 eyes with RNFL-based alerts, 5 archetypes covered 296 eyes (91%), whereas 28 eyes (9%) with alerts were unclassifiable. Of the 296 classifiable eyes, 177 eyes (60%) fit a single archetype, whereas 119 eyes (40%) fit two archetypes in various combinations (Figure 3).

**Figure 4:**
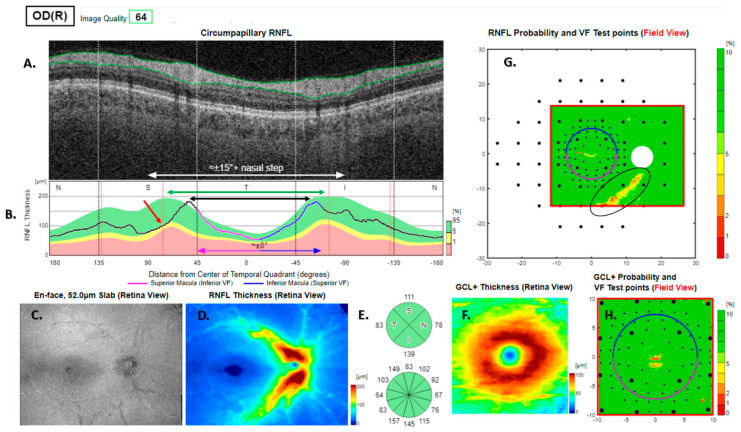
Type 1 RNFL thickness deficit alert from a right eye with a normal visual field and a narrow angle between the arcuate nerve fibre ridges ((**B**) double-headed black arrow). The green arrow shows the template distance between the RNFL peaks. A local minimum superior of the optic disc (red arrow) appears to be responsible for the thickness deficit alert, (**G**) but since the visual field was normal, the peripapillary RNFL distribution must been balanced by the higher-than-normal thickness of the adjacent RNFL. The peripapillary retinal nerve fibre layer (RNFL (**A**)) and its thickness curve in ((**B**) black, blue and purple); N, nasal; S, superior; T, temporal; I, inferior quadrants; 0 degrees representing the temporal horizontal meridian. Red vertical lines (**B**) show the location of the superior and inferior arcuate trunk vessels. The peaks of the superior and inferior arcuate nerve fibre ridges or the template or reference curve are close to the innermost red lines. An en face slab of the inner retina shows the RNFL analysis (**C**), RNFL thickness map (**D**), and RNFL sector analysis (**E**). The macular ganglion cell and inner plexiform layer (GC-IPL) thickness map is shown on (**F**). The RNFL probability map is shown on (**G**) and the GC-IPL probability map on (**H**). In (**G+H**) green colour indicates that thickness is within the best 95% of healthy subjects at a given test location. For other colours, see colour bar next to (**G+H**). VF, visual field. GCL+ above = GC-IPL.

The five archetypes were as follows:

The Type (1) Narrow RNFL angle, found in 70 eyes (11%), is an alert that arises from the temporal nerve fibre ridges being separated by a shorter angular distance than the reference (Figure 4, panel B and panel D). This deviation from the norm leads to a nominal RNFL thickness deficit where the reference curve has its highest thickness. Given that the visual field was normal in the investigated eyes, the nominal deficit must have been fully compensated by the extra RNFL mass that was found closer to the temporal horizontal meridian. The alerts extended from the physiological blind spot, were arcuate and wedge-shaped, and followed the anatomical RNFL distribution.

**Figure 5:**
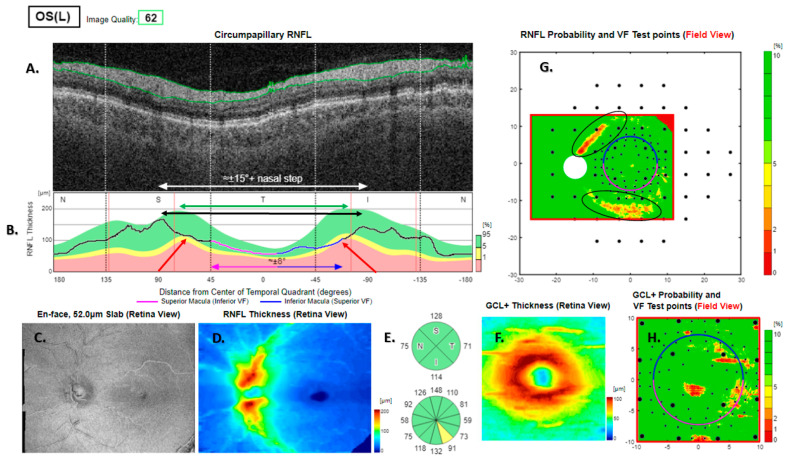
Type 2 RNFL thickness deficit alerts from a healthy left eye. The angle between the temporal nerve fibre ridges is wide (black double-headed arrow) compared to the template (green double-headed arrow). This results in nominal thickness deficits (red arrows) superior and inferior of the disc, that happen to be balanced by thick adjacent nerve fibre sectors (**B**). The peripapillary retinal nerve fibre layer (RNFL (**A**)) and its thickness curve in ((**B**) black, blue and purple); N, nasal; S, superior; T, temporal; I, inferior quadrants; 0 degrees representing the temporal horizontal meridian. Red vertical lines (**B**) show the location of the superior and inferior arcuate trunk vessels. The peaks of the superior and inferior arcuate nerve fibre ridges or the template or reference curve are close to the innermost red lines. An en face slab of the inner retina shows the RNFL analysis (**C**), RNFL thickness map (**D**), and RNFL sector analysis (**E**). The macular ganglion cell and inner plexiform layer (GC-IPL) thickness map is shown on (**F**). The RNFL probability map is shown on (**G**) and the GC-IPL probability map on (**H**). In (**G+H**) green colour indicates that thickness is within the best 95% of healthy subjects at a given test location. For other colours, see colour bar next to (**G+H**). VF, visual field. GCL+ above = GC-IPL.

The Type (2) Wide RNFL angle, found in 81 eyes (13%), is an alert that arises from the temporal nerve fibre ridges being separated by a large angular distance compared to the reference (Figure 5, panels B and D). The alerts are comparable to Type 1 defects, but they are located closer to the fovea.

**Figure 6:**
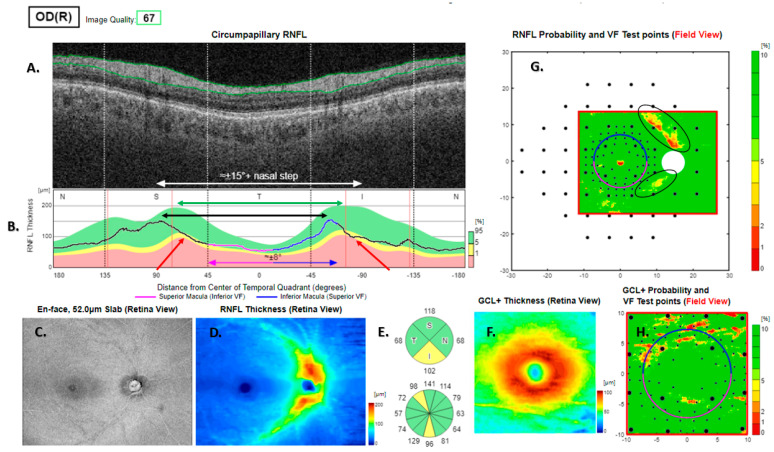
Type 3 RNFL thickness deficit alert from a healthy right eye. The temporal nerve fibre ridges are rotated 15 degrees clockwise relative to the template (green and black double-headed arrows in (**B**)), giving rise to arcuate alerts in (**G**). This is due to the thickness deficit at the red arrows in (**B**). The peripapillary retinal nerve fibre layer (RNFL (**A**)) and its thickness curve in ((**B**) black, blue and purple); N, nasal; S, superior; T, temporal; I, inferior quadrants; 0 degrees representing the temporal horizontal meridian. Red vertical lines (**B**) show the location of the superior and inferior arcuate trunk vessels. The peaks of the superior and inferior arcuate nerve fibre ridges or the template or reference curve are close to the innermost red lines. An en face slab of the inner retina shows the RNFL analysis (**C**), RNFL thickness map (**D**), and RNFL sector analysis (**E**). The macular ganglion cell and inner plexiform layer (GC-IPL) thickness map is shown on (**F**). The RNFL probability map is shown on (**G**) and the GC-IPL probability map on (**H**). In (**G+H**) green colour indicates that thickness is within the best 95% of healthy subjects at a given test location. For other colours, see colour bar next to (**G+H**). VF, visual field. GCL+ above = GC-IPL.

The Type (3) Rotational RNFL deviation, seen in 23 eyes (4%), is a false field defect prediction that occurs when the two temporal RNFL ridges are shifted in the same rotational direction compared to the reference RNFL thickness curve (Figure 6). This results in the production of one or two arcuate alerts located at unequally angular distances from the horizontal meridian (Figure 6).

**Figure 7:**
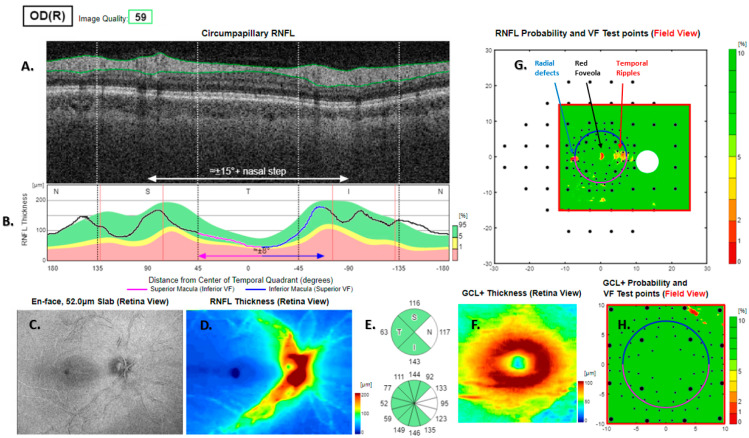
Type 4 RNFL thickness deficit alert from a healthy right eye. Thickness deficit alerts (**G**) are associated with nominally subnormal thickness at the temporal meridian compensated by thicker than normal RNFL elsewhere (**B**) and was associated with characteristic visual field alerts (temporal ripples, red fovea and radial defect, (**G**)). The peripapillary retinal nerve fibre layer (RNFL (**A**)) and its thickness curve in ((**B**) black, blue and purple); N, nasal; S, superior; T, temporal; I, inferior quadrants; 0 degrees representing the temporal horizontal meridian. Red vertical lines (**B**) show the location of the superior and inferior arcuate trunk vessels. The peaks of the superior and inferior arcuate nerve fibre ridges or the template or reference curve are close to the innermost red lines. An en face slab of the inner retina shows the RNFL analysis (**C**), RNFL thickness map (**D**), and RNFL sector analysis (**E**). The macular ganglion cell and inner plexiform layer (GC-IPL) thickness map is shown on (**F**). The RNFL probability map is shown on (**G**) and the GC-IPL probability map on (**H**). In (**G+H**) green colour indicates that thickness is within the best 95% of healthy subjects at a given test location. For other colours, see colour bar next to (**G+H**). VF, visual field. GCL+ above = GC-IPL.

The Type (4) Temporal ripples, seen in 97 eyes (16%), are small alerts, concentric with the fovea, seen on either side of the centre of the visual field centre, mostly near the physiological blind spot (Figure 7). Their source was not identified. Of the 97 eyes, 51 (53% of the eyes with this feature and 8% of all the eyes) showed predominantly temporal ripples, whereas 46 (47% of the eyes with this feature and 7% of all the eyes) also displayed RNFL-based alerts (Figure 7).

**Figure 8:**
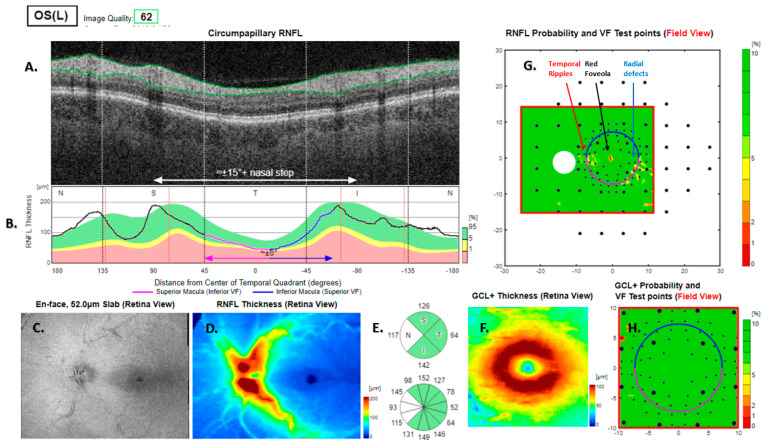
Type 5 RNFL thickness deficit alert from a left eye with a normal visual field. The angle between the temporal nerve fibre ridges is large (**B**). Thickness deficit alerts, as projected on the visual field, are associated with a nominal thickness deficit at the temporal meridian (**B**), which is balanced by thick RNFL elsewhere. The alert types are radial defects, centre artefacts, and temporal ripples. The peripapillary retinal nerve fibre layer (RNFL (**A**)) and its thickness curve in ((**B**) black, blue and purple); N, nasal; S, superior; T, temporal; I, inferior quadrants; 0 degrees representing the temporal horizontal meridian. Red vertical lines (**B**) show the location of the superior and inferior arcuate trunk vessels. The peaks of the superior and inferior arcuate nerve fibre ridges or the template or reference curve are close to the innermost red lines. An en face slab of the inner retina shows the RNFL analysis (**C**), RNFL thickness map (**D**), and RNFL sector analysis (**E**). The macular ganglion cell and inner plexiform layer (GC-IPL) thickness map is shown on (**F**). The RNFL probability map is shown on (**G**) and the GC-IPL probability map on (**H**). In (**G+H**) green colour indicates that thickness is within the best 95% of healthy subjects at a given test location. For other colours, see colour bar next to (**G+H**). VF, visual field. GCL+ above = GC-IPL.

The Type (5) Radial defects, seen in 144 eyes (23%), typically spans the edge of the macular visual field at a distance of 8 ± 4 degrees from the centre of the visual field (Figure 8). Their location and direction suggest that they may be related to the periodicity of the peripapillary nerve fibre layer thickness curve associated with the intercalation of the RNFL with the peripapillary vessel segments. This phenomenon is hypothetically influenced by the method used to interpolate RNFL thickness, where the layer is interspersed with large vessels. This artefact is most prominent within 8 degrees of the centre of the visual field. Of the 144 eyes, 71 (49% of the eyes in the group and 12% of all the eyes) showed predominantly radial defects, whereas 73 (51% of the eyes in the group and 12% of the total eyes) also displayed other prominent RNFL-based alerts (Figure 8).

After the elimination of 368 eyes with normal visual field predictions, 245 eyes with GC-IPL based visual field alerts without corresponding visual field defects fitted a classification with four archetypes based on thickness distribution, diameter, and foveal thickness (Figure 1, panel H).

**Figure 9:**
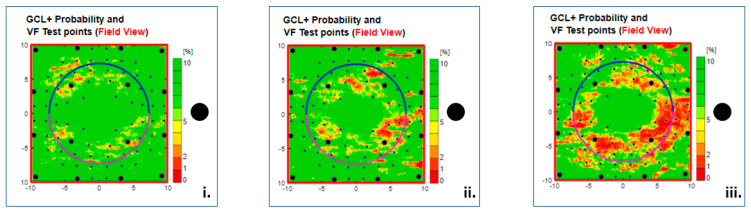
Type 6 GC-IPL-related thickness deficit alerts in three healthy right eyes. Perifoveal patterns of annular clutter are seen in all three eyes with increasing density from left to right, from rough outline (**i**), over half-circle (**ii**) to full circle (**iii**). The black dots to the right of the scale indicate the physiological blind spots. GC-IPL, ganglion cell–inner plexiform layer. VF, visual field. GCL+ above = GC-IPL.

The Type (6) Annular clutter, seen in 77 eyes (12%), is an alert with an irregular or incomplete annular foveola-centred pattern and an intact visual field centre. Based on the variations in the continuity, extent, and depth of the predicted scotoma, the artefact was subdivided into severity levels: 1, 2, and 3 (Figure 9).

**Figure 10:**
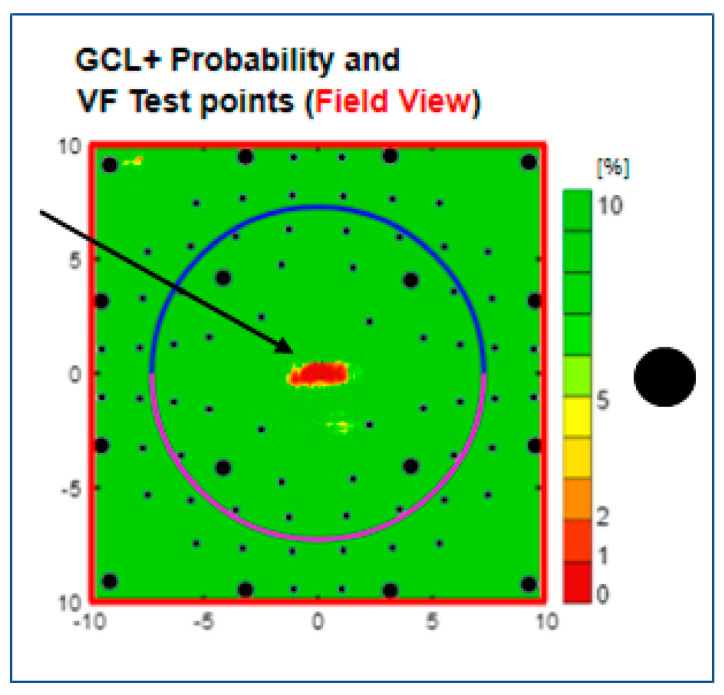
Type 7 GC-IPL related thickness deficit alert from a healthy right eye showing a central defect. The black dot to the right of the scale indicates the physiological blind spot. GC-IPL, ganglion cell–inner plexiform layer. VF, visual field. GCL+ above = GC-IPL.

Type (7) Central spot, seen in 38 eyes (6%), is an alert in the centre of the visual field (Figure 10) that resembles the RNFL-based red foveola phenomenon described above.

**Figure 11:**
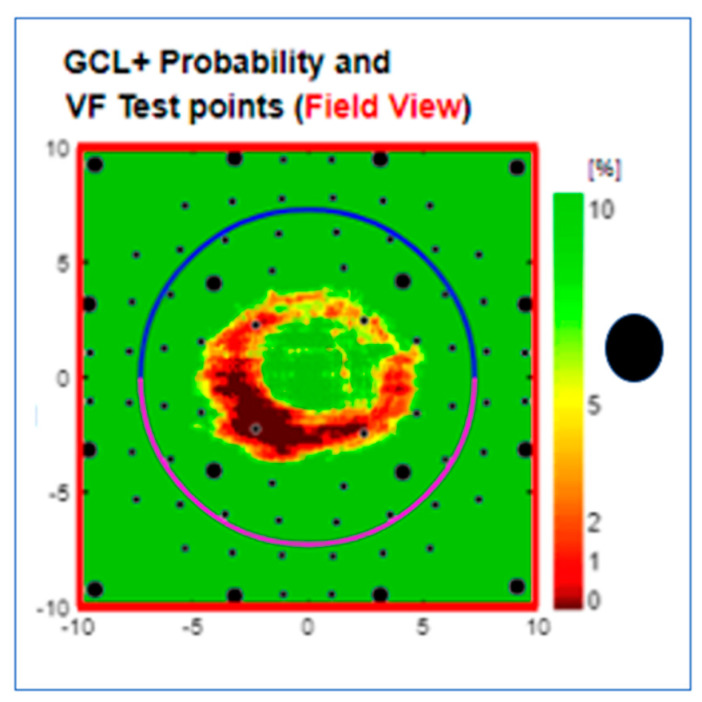
Type 8 GC-IPL-related doughnut-shaped thickness deficit alert from a right eye with a normal visual field. The black dot to the right of the scale indicates the location side of the physiological blind spot. GC-IPL, ganglion cell–inner plexiform layer. VF, visual field. GCL+ above = GC-IPL.

The Type (8) Doughnut, seen in 10 eyes (2%), has the shape of a contiguous pericentral visual field defect at a short distance from the foveal centre (Figure 11).

**Figure 12:**
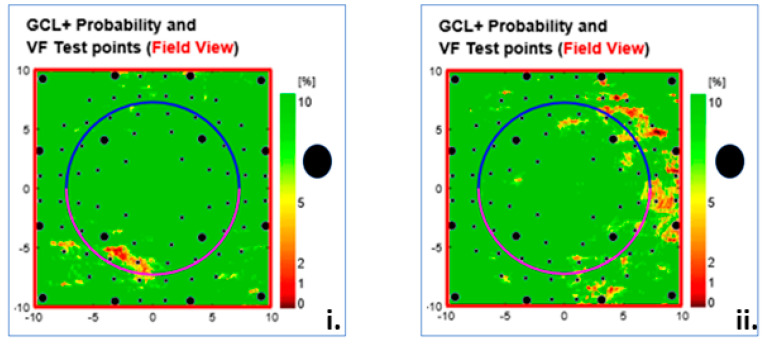
Type 9 GC-IPL-related thickness deficit alerts from two healthy right eyes showing different degrees of irregularly located alerts in indistinct patterns, from mild (**i**) to severe (**ii**). The black dots indicate the location of the physiological blind spot. GC-IPL, ganglion cell–inner plexiform layer. VF, visual field. GCL+ above = GC-IPL.

The Type (9) Local clutter, seen in 120 eyes (20%), presents as irregular clusters of defects around the centre of the visual field (Figure 12). The artefact was subdivided into severity levels 1 and 2 (Figure 12).

The eyes with RNFL-based alert type 1 and unclassifiable RNFL-based alerts group were longer and more myopic than the eyes without alerts (Table 3). Likewise, the eyes with GC-IPL-based alerts type 6 and 9 were longer and more myopic than the eyes without such defects, whereas the eyes with type 7 alerts were shorter and the eyes with type 8 alerts were shorter and more hypermetropic (Table 4). Furthermore, the increasing prominence of the annular and local clutter alerts (types 6 and 9) were associated with longer and more myopic eyes (Table 5).

We compared the eyes with no alerts at all to the eyes with both RNFL and GC-IPL alerts, only RNFL alerts, and only GC-IPL alerts. We found that the eyes with both RNFL and GC-IPL alerts were more myopic and had a longer axial length than the eyes with no alerts at all (Table 6). Furthermore, the eyes with only GC-IPL had a longer axial length than the eyes with no alerts at all. No significant difference in IOP, MD, or age was seen between these groups.

**Figure 13:**
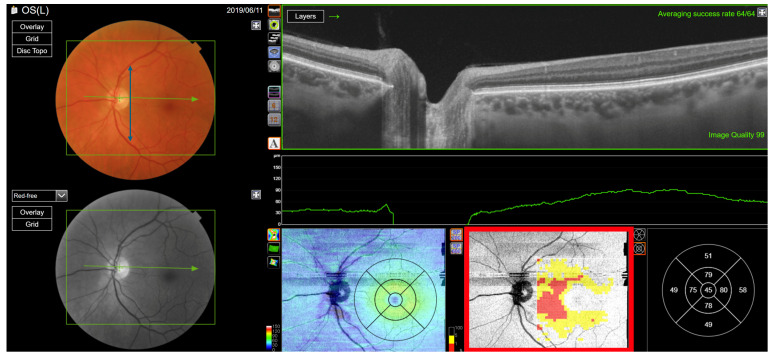
Ganglion cell layer analysis of a healthy left eye with a wide macula. The vessel separation angle is wide (blue double arrow). The ganglion cell layer is thin at the centre of macula, except at the rim of the fovea (red rectangle).

**Figure 14:**
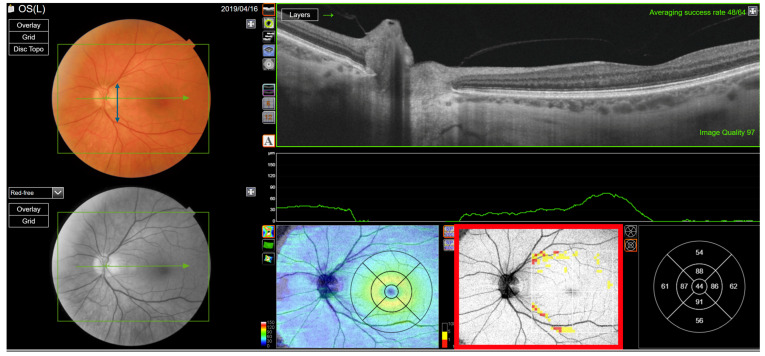
Ganglion cell layer analysis of a healthy left eye with a narrow macula. The vessel separation angle is narrow (blue double arrow). The ganglion cell layer thickness is normal at the centre of macula, except at the border of the vessels (red rectangle).

A correlation between RNFL- and GC-IPL-based classifications was seen in that the 26 (34%) out of the 77 eyes with type 6 annular clusters were RNFL type 2 eyes with a wide RNFL ridge separation angle (Table 7), thus showing that when the separation angle is wide, the macula tends to be thinner (Figure 13). When the separation angle is narrow, the centre thickness of the macular centre tends to be normal compared to the reference group (Figure 14).

## 4. Discussion

This study of an automated OCT-based method of identifying retinal nerve fibre and ganglion cell layer deficits in healthy human eyes found that anatomical variations from a standard reference template explained more than 90% of the deficit alerts in the eyes with normal visual fields. The principal form of variation was found to be in the angle between the arcuate nerve fibre ridges that encircle the macula, in the thickness and width of the macula, or in a combination thereof.

In clinical practice, alerts that are likely not accompanied by visual field defects such as the red foveola alert (Figure 2), RNFL deficit alert type 4 (Figure 7), or GC-IPL deficit alert type 7 (Figure 10) can be identified and recognised with no suspicion for glaucoma. However, other types of RNFL and GC-IPL deficit alerts can be challenging for the practician to differentiate from absolute visual field defects. These deficit alerts often mimic deep arcuate scotomas, which in a glaucoma screening context means that they cannot be dismissed by raising the threshold for referral.

In a glaucoma screening context, the elimination of glaucoma suspicion should be fast and effective. This study indicates that efficiency can be increased through consideration of a limited number of anatomical variants. Adjusting for the peripapillary RNFL ridges separation angle and axial length might reduce but not eliminate the number of deficit alerts. Even if glaucoma screening is limited to specific high-risk groups, the largest fraction will not have glaucoma. Robust recognition of normality is therefore vital for cost-effectiveness.

Anatomical subtype identification will require that sufficient inner retina has been preserved to identify characteristics such as the angle between the nerve fibre ridges. If they are absent, though, the presence of glaucoma or some other severe manifest disease should not be in doubt.

Although no definite anatomical explanation could be found in 8% of the RNFL-based alerts, the patterns in question were not easily confused with those seen in glaucoma patients. These eyes were myopic with longer axial length, with possible different retinal anatomical variations that deviated from the reference group.

The insights derived from this study add to a prior suggestion that, in a crude assessment of the RNFL distribution in a given eye, it may be assumed that if the eye is healthy, a nominal deficit in one sector can be compensated by a nominal surplus in an adjacent sector [4]. It has previously been shown that there are interindividual variations in the angular separation of the peripapillary RNFL ridges, ranging from 64 to 91 degrees [5]. There is also a correlation between the location of the major temporal vessels and the temporal RNFL ridges, but with considerable interindividual deviations [6,7]. In line with this, a long distance between the foveola and the optic disc is associated with a narrow angle between the RNFL ridges and vice versa [3]. Adjusting for these anatomical factors in the Hood Report might reduce the deficit alerts.

There is a high correlation between the angular separation of the peripapillary RNFL ridges of fellow eyes [5]. Thus, there should be a fair chance of using fellow eyes as references for each other in a glaucoma screening programme. If one eye has an area with thinner peripapillary RNFL thickness compared to the fellow eye, it should give the practician a suspicion for abnormality.

Previous studies have found rates of thickness deficit alerts that approach those seen in the present study, and the rates were found to increase with increasing myopia and extreme fovea–disc distances [8,9,10]. Unlike the present study, none of the prior studies were population-based or had the same range in age.

Previous studies have found an association between shorter or longer axial lengths and the increased prevalence of RNFL- and GC-IPL-based thickness deficit alerts [9,11,12]. These correlations are likely to be explained by the anatomical variations identified in the present study. We observed that eyes with RNFL and GC-IPL alerts were significantly more myopic than the eyes with no alerts. Consequently, to minimise false alerts and enhance the specificity of glaucoma detection using OCT probability maps in myopic eyes, the implementation of a healthy myopic normative database is essential [13].

Thickness deficit alerts related to rotational shift were occasionally found in this study to be associated with a head tilt. However, in most cases, the abnormality was symmetric, showing that true variation in the angle between the disc and the fovea does occur in healthy subjects.

A red foveola phenomenon was found on 73% of the RNFL-based visual field predictions. This is a paradox, given that common standards of automated perimetry omit the foveal centre from assessment. We propose that a corresponding part of the visual field-projected thickness map be left blank.

The GC-IPL-based visual field prediction should obviously fit the RNFL-based map [14] within the area covered by both, but this is not always the case [15,16]. Technically, the large tissue volume of the RNFL and its high optical contrast on OCT may seem to favour a RNFL-based visual field prediction map [17,18].

In our study, the thicknesses of the RNFL and the GC-IPL were compared to the instrument manufacturer’s database, which contains 137 and 164 3-dimensional discs and macula scans, respectively, of healthy persons aged 19–84 years, but included only axial lengths between 22 and 26 mm and spherical equivalent refraction between +3.00 D and −6.00 D. It is expected to find retinal anatomical differences between eyes with 22 mm vs. 26 mm; therefore, adjusting the reference database to the examined eye is necessary. Chua et al. have previously shown that when applying a multivariate normative database with compensated RNFL thickness for multiple demographic and anatomic variation, the deviation alerts were reduced [19]. Thus, there may be advantages to including a larger, broader, adjusted and more in-depth characterised set of reference data.

A limitation of this study is that it only included a single automated perimetry examination, as opposed to repeated testing that can account for training effects. A single procedure in untrained subjects is the relevant comparator, however, in a screening scenario.

The peak prevalence of glaucoma is at age 85–89 years [20]. Our study population is considerably younger, having a median age of 59 years and an age range from 30 to 80 years. Thus, it does not cover the need for normative data, but the types of anatomical variation that give rise to thickness deficit alerts are likely, in our opinion, to be the same over a large age range. It should also be noted that the study included only twins, who may not be fully representative of the non-twin population.

This study used single-visit data to examine the relation between RNFL topography analysis and visual field characteristics. Longitudinal OCT analyses may have advantages in detecting glaucoma; although, one such study found that over 10 years, the off-set between the glaucoma patients and healthy subjects remainder was stable [21].

This study focussed on single alerts in healthy eyes via a method developed for the detection of glaucoma. The identification of glaucoma can obviously be based on a combinations of alerts and refined decision rules [22]. It has been suggested that retinal tissue loss may precede glaucomatous visual field loss, but the evidence does not support this claim [23].

## 5. Conclusions

In conclusion, anatomical variation in the retinal nerve fibre and ganglion cell layer distribution was the source of different types of thickness deficit alerts in the eyes with normal visual fields in this study. The healthy eyes with RNFL and GC-IPL deviation alerts were significantly more myopic with longer axial length than the healthy eyes with no alerts. Automated analysis can potentially be improved through the use of reference data that fits the inherent anatomical phenotype of a given eye.

## Figures and Tables

**Table 1:** Characteristics of study population.

Variable	Number/Median	Number of Eyes/Range
All participants	360	720
Excluded (%)	33 (9%)	107 (15%)
Included (%)	327 (91%)	613 (85%)
Monozygotic twins (%)	171 (52%)	320 (52%)
Dizygotic twins (%)	156 (48%)	293 (48%)
Unpaired twins (%)	29 (9%)	44 (7%)
Unpaired monozygotic	19 (6%)	26 (4%)
Unpaired dizygotic (%)	10 (3%)	18 (3%)
Male (%)	146 (45%)	277 (45%)
Female (%)	181 (55%)	336 (55%)
Age, years	59	30 to 80
Refraction right eye, D	0	−10.75 to +7.50
Refraction left eye, D	0	−10.50 to +8.00
Visual acuity right eye, EDTRS letters	89	70 to 100
Visual acuity left eye, EDTRS letters	89	70 to 99
IOP right eyes, mmHg	14	8 to 24
IOP left eyes, mmHg	14	7 to 24
Mean defect right eye, dB	1.80	−1.5 to +5.9
Mean defect left eye, dB	1.80	−1.5 to +5.9

ETDRS, Early Treatment Diabetic Retinopathy Study. IOP, intraocular pressure.

**Table 2:** Type of visual field-projected tissue defect alert.

Classification	Origin	Number of Eyes (Total 613)	Twin A or Unpaired (Total 324)
No alert	RNFL	289 (47%)	163 (50%)
Type (1) Narrow RNFL angle	RNFL	70 (11%)	29 (9%)
Type (2) Wide RNFL angle	RNFL	81 (13%)	41 (13%)
Type (3) Rotational RNFL deviation	RNFL	23 (4%)	9 (3%)
Type (4) Temporal ripples	RNFL	51 (8%)	30 (9%)
Type (5) Radial defects	RNFL	71 (12%)	36 (11%)
Unclassifiable	RNFL	28 (5%)	16 (5%)
No alert	GC-IPL	368 (60%)	188 (58%)
Type (6) Annular clutter	GC-IPL	77 (12%)	45 (14%)
Type (7) Central spot	GC-IPL	38 (6%)	22 (7%)
Type (8) Doughnut	GC-IPL	10 (2%)	6 (2%)
Type (9) Local clutter	GC-IPL	120 (20%)	63 (19%)
No alert	RNFL + GC-IPL	222 (36%)	119 (37%)
Alerts in total	RNFL + GC-IPL	391 (64%)	205 (63%)

RNFL, retinal nerve fibre layer. GC-IPL, ganglion cell layer–inner plexiform layer.

**Table 3:** RNFL-based tissue defect alerts in relation to axial length and refraction.

RNFL Classification Groups	Axial Length (mm)		Spherical Equivalent Refraction (D)	
	Median	*p*-Value	Median	*p*-Value
No alert	23.43	reference	+0.25	reference
Narrow RNFL angle (type 1)	24.44	<0.01	−1.37	<0.01
Wide RNFL angle (type 2)	23.54	0.32	+0.13	0.97
Rotational RNFL deviation (type 3)	23.92	0.07	−0.50	0.38
Temporal ripples (type 4)	23.40	0.99	0.00	0.85
Radial defects (type 5)	23.72	0.13	−0.25	0.21
Unclassifiable	24.64	<0.01	−2.50	<0.01

RNFL, retinal nerve fibre layer.

**Table 4:** GC-IPL-based tissue defect alerts in relation to axial length and refraction.

GC-IPL Classification	Axial Length		Spherical Equivalent Refraction (D)	
	Median	*p*-Value	Median	*p*-Value
No alert	23.42	Ref.	0.00	ref
Annular clutter (type 6)	23.93	<0.01	−0.25	0.05
Central spot (type 7)	22.81	0.03	+0.37	0.25
Doughnut (type 8)	22.99	0.03	+1.5	0.04
Local clutter (type 9)	24.20	<0.01	−0.44	<0.01

GC-IPL, ganglion cell–inner plexiform layer.

**Table 5:** Minor GC-IPL-based visual field-projected tissue defect alerts in relation to axial length and refraction.

Group	Subgroup	Axial Length (mm)		Spherical Equivalent Refraction (D)	
		Median	*p*-Value	Median	*p*-Value
No alert	-	23.42	1	0	1
Type 6	Annular clutter-total	23.93	<0.01	−0.25	0.03
	Level i	23.65	0.14	0.31	0.5
	Level ii	23.72	0.01	−0.37	0.01
	Level iii	24.30	<0.01	−0.25	0.01
Type 9	Local clutter-total	24.20	<0.01	−0.44	<0.01
	Level i	24.14	<0.01	−0.25	0.02
	level ii	24.90	<0.01	−1.38	<0.01

The table covers subtypes 6 1–3 and 9 1 and 2. Comparison made using the Wilcoxon test. GC-IPL ganglion cell layer–inner plexiform layer.

**Table 6:** Comparison of eyes with no alerts and with alerts in relation to axial length and refraction.

Type of Alerts	Axial Length		Spherical Equivalent Refraction (D)	
	Median	*p*-Value	Median	*p*-Value
No alerts at all	23.36	Ref.	+0.25	ref
RNFL and GC-IPL alerts	24.09	<<0.01	−0.25	<<0.01
Only RNFL	23.43	0.38	+0.18	0.39
Only GC-IPL	23.97	<0.01	+0.06	0.16

RNFL, retinal nerve fibre layer. GC-IPL, ganglion cell–inner plexiform layer.

**Table 7:** Coincidence of RNFL- and GC-IPL-based visual-field projected tissue defect alert.

RNFL\GC-IPL	Annular Clutter (Type 6)	Central Spot (Type 7)	Doughnut (Type 8)	Local Clutter (Type 9)
No alert	15 (19%)	17 (45%)	3 (30%)	31 (26%)
Narrow RNFL angle (type 1)	7 (9%)	4 (11%)	1 (10%)	32 (27%)
Wide RNFL angle (type 2)	26 (34%)	3 (8%)	1 (10%)	16 (13%)
Rotational RNFL deviation (type 3)	4 (5%)	0 (0%)	0 (0%)	9 (8%)
Temporal ripples (type 4)	6 (8%)	7 (18%)	5 (50%)	9 (8%)
Radial defects (type 5)	8 (10%)	5 (13%)	0 (0%)	15 (13%)
Unclassifiable	11 (14%)	2 (5%)	0 (0%)	8 (7%)
In total	77 (100%)	38 (100%)	10 (100%)	120 (100%)

RNFL, retinal nerve fibre layer. GC-IPL, ganglion cell–inner plexiform layer.

## Data Availability

The original contributions presented in this study are included in the article. Further inquiries can be directed to the corresponding author(s).
